# Introduction to the multi-author review on macular degeneration

**DOI:** 10.1007/s00018-019-03418-5

**Published:** 2020-01-02

**Authors:** Anu Kauppinen

**Affiliations:** Faculty of Health Sciences, School of Pharmacy, University of Eastern Finland, 1627, 70211 Kuopio, Finland

**Keywords:** Age-related macular degeneration, Senescence, Immune cells, Epigenetics, Hypoxia, Lipocalin

## Abstract

Prolonged life expectancies contribute to the increasing prevalence of age-related macular degeneration (AMD) that is already the leading cause of severe vision loss among the elderly in developed countries. In dry AMD, the disease culminates into vast retinal atrophy, whereas the wet form is characterized by retinal edema and sudden vision loss due to neovascularization originating from the choroid beneath the Bruch’s membrane. There is no treatment for dry AMD and despite intravitreal injections of anti-vascular endothelial growth factor (VEGF) that suppress the neovessel formation, also wet AMD needs new therapies to prevent the disease progression and to serve patients lacking of positive response to current medicines. Knowledge on disease mechanisms is a prerequisite for the drug development, which is hindered by the multifactorial nature of AMD. Numerous distinguished publications have revealed AMD mechanisms at the cellular and molecular level and in this multi-author review, we take a bit broader look at the topic with some novel aspects.

Age-related macular degeneration (AMD) is an ocular disease localizing mainly at the macular area, which is responsible for central and sharp vision at the posterior part of the eye. The disease impairs, e.g. abilities to read, dial numbers, recognize faces and thereby, it severely compromises independency in daily tasks [3]. Loss of photoreceptors is preceded by the dysfunctionality and death of retinal pigment epithelium (RPE) cells that normally form a solid single-cell layer between photoreceptors and the Bruch’s membrane. Reasons for the RPE degeneration are diverse but immune dysfunction, oxidative stress, mitochondrial damage, disturbed proteostasis, complement activation, and inflammation constantly recur in publications [1, 3, 6, 8–12, 16, 19]. Despite the central role of the RPE, cells of innate and adaptive immunity are also involved in the pathogenesis of AMD. Chemokines secreted upon retinal damage recruit resident microglia as well as systemic leukocytes to the subretinal area [3]. Here, Verena Behnke et al. provide insight into the role of immune cells in AMD.

In a systematic meta-analysis with 16 identified risk factors, aging, current smoking, cataract surgery, and a family history were strongly associated with late AMD that can be divided into dry and wet forms [4, 5]. Also, cardiovascular disease-related factors, such as hypertension, and a history with cardiovascular disease were observed as significant risk factors for AMD [5]. Aging is a strong prerequisite for AMD, the prevalence of which is low in people under 60 years [20]. Aging is associated with cellular senescence [7] that is contemplated in this multi-author review (MAR) by Janusz Błasiak in relation to premises of RPE cells upon stressful conditions.

In addition to environmental risk factors, genetic predisposition is associated with AMD and especially immune modulation and complement system are represented among susceptibility genes [15]. It has been observed that the role of genetic factors ranges from 46 to 71% in AMD [17, 20]. Nowadays, it is known that in addition to inherited genes, epigenetic modulation can change gene activity and expression without altering the DNA sequence [21]. Contribution of epigenetics is a very current topic in which interest also in relation to AMD is increasing, and that subject is covered here by Maria Gemenetzi and Andrew Lotery.

Intracellular accumulation of lipofuscin in lysosomes and extracellular drusen deposits between the RPE and the Bruch’s membrane are the first clinical signs observed upon diagnosis of AMD [14]. Drusen and retinal edema are included in factors resulting in hypoxia found in AMD eyes [18]. The role of hypoxia in ocular neovascularization has recently been reviewed elsewhere [2] and in this MAR, hypoxia is considered from the perspective of gene therapy by Parviz Mammadzada et al.

Lipocalin-2 (LCN-2) belongs to the lipocalin protein family, the members of which share evolutionarily conserved eight-stranded antiparallel β-sheet “barrel” structure with capacity to bind small hydrophobic ligands [13]. LCN-2 is associated with infections, acute inflammation, as well as several chronic diseases [13]. Its role in AMD has not gained much attention but is now being compiled by Sayan Ghosh et al.

RPE cells are in the central role in the pathogenesis of AMD but several important players beyond them must also be taken into account when considering this multifactorial disease. This multi-author review presents AMD from other perspectives deepening the understanding on its pathogenesis and providing views for new therapy options.
